# Enhancing Strength and Quantifying Sustainability of Building Blocks Manufactured by Geopolymerization

**DOI:** 10.3390/ma17040964

**Published:** 2024-02-19

**Authors:** Khadija Mawra, Khuram Rashid, Muhammad Irfan-ul-Hassan, Idrees Zafar, Mounir Ltifi

**Affiliations:** 1 Department of Architectural Engineering and Design, Faculty of Civil Engineering, University of Engineering and Technology, Lahore 54890, Pakistan; 2 Department of Civil Engineering, University of Engineering and Technology, Lahore 54890, Pakistan; 3 Department of Civil Engineering, Imam Mohammad Ibn Saud Islamic University, Riyadh 11432, Saudi Arabia; 4 Department of Civil Engineering, National Engineering School of Gabes, University of Gabes, Gabes 6072, Tunisia

**Keywords:** geopolymer, pressured catalysis, mineral, environmental and economic indices, sustainability

## Abstract

Enhancing the strength of fly ash (FA)-based geopolymer by increasing the alkaline activator content is a costly and unsustainable technique. Therefore, this work was designed to reduce the activator by employing the pressured catalysis (PC) technique, coupled with the use of minerals that have filler and occupying effects. The main objective was to enhance the strength of the mix with a lower alkaline-to-precursor (A/P) ratio and create a sustainable, load-bearing building block from it. Initially, the compressive strength of the FA-based geopolymer was investigated experimentally by varying sodium silicate to sodium hydroxide and A/P ratios with ambient and hot curing. Afterward, PC was applied to the optimized proportion of constituents, and a significant increase in strength (9.6 to 20.0 Mpa) was observed at a 0.25 A/P ratio. By adding clay and dune sand (DS), the compressive strength was 19.5 and 40.4 Mpa at an A/P of 0.25 and 0.16, respectively. The strength gain mechanism was evaluated at the molecular and micro levels by conducting FTIR and SEM analyses. The environmental and economic indices and strength indicated the high sustainability of DS-based geopolymers compared to analogous blocks. The environmental and economic benefits of 23.9% reduced CO_2_ emissions and 24.2% less cost were provided by the DS-based block compared to the FA–clay-based block. A DS-based geopolymer obtains strength at a low A/P due to its occupying effect and results in sustainable building blocks.

## 1. Introduction

Fly ash (FA) has been used in the production of sustainable cementless concrete by successfully activating FA through alkali activators and instigating its geo-polymerization [1,2]. However, these activators are hazardous, corrosive, and costly; therefore, attempts are needed to reduce their dosage and investigate alternate means to facilitate the process of geo-polymerization. Dune sand (DS) is one such coupling material, whose chemical composition has been widely studied and considered unfit as a conventional, cement-based construction material. However, its utilization in geopolymers and behavior regarding strength gains need to be investigated. Moreover, a novel technique is required, which can reduce the activator amount without reducing the strength.

Recently, in geopolymers, various novel materials have been utilized, like waste glass [3], red mud [4], and nanomaterials [5], among other rich aluminosilicate materials, like FA, slag, bagasse ash, and clay [6,7]. These are activated in alkaline environments (a combination of sodium silicate (Na_2_SiO_3_) and sodium hydroxide (NaOH)), which results in the formation of tetrahedral structures, which are responsible for the development of strength [8]. Thus, geopolymer exhibits a performance like conventional, cement-based construction materials [9,10]. The strength in geopolymers was achieved by the formation of sodium aluminosilicate hydrate (N-A-S-H) and calcium aluminosilicate hydrate (C-A-S-H) gels, based on the use of low or high calcium-containing precursors. Both gels are formed after leaching Al, Si, and Ca from the precursor in an alkaline environment [4,11], where the dissolution of these species depends upon the molar concentration of NaOH solution, chemical composition (silica and other constituents), and the curing temperature [11]. The liquid-to-solid or alkaline-to-precursor (A/P) ratios are a decisive factor in the development and strength gain of geopolymers. Along with these ratios, the concentration of NaOH, Na_2_SiO_3_/NaOH (SS/SH) ratio, and curing temperature also contribute to the strength gain. Therefore, these factors must be investigated due to the versatile behavior of the FA. Additionally, what would happen if FA was coupled with any supplementary cementitious or non-cementitious materials is also an important area on which to focus. 

Lots of studies are available on FA-based geopolymer, and the above-mentioned parameters (A/P ratio, Na_2_SiO_3_/NaOH ratio, and curing temperature) have been studied experimentally as well as by using artificial intelligence [12]. The influencing parameter on the strength of geopolymers is the A/P ratio; the more the activator is used, the higher the strength, and from a database of 90 sources, the value of A/P ratio varies from 0.30 to 0.92 [12,13]. In most of the studies, the A/P is more than 0.4., and this has also been verified by the histogram mentioned in Appendix A. A lower activator value means there are unreacted precursor particles, and if the dosage of the activator is higher, then there will be congestion in the paste and the strength will be reduced [14,15]. Similarly, another parameter that influences the strength of the geopolymer is the SS/SH ratio. The SiO_2_ content from Na_2_SiO_3_ balances the silica modulus, and its higher value may behave similarly to the congestion behavior of a high A/P ratio. Therefore, different values have been used for the SS/SH in the range of 2.0–8.0. From more than 70 database values, the SS/SH varies from 2.0 to 3.0 (Appendix A). The histogram for curing temperature has also been plotted in Appendix A, and it can easily be concluded that, for an FA-based geopolymer, the curing temperature lies from 60 to 100 °C. At the current stage, a challenge in FA geopolymer research is lowering the alkali requirements through the incorporation of different geo-polymerization acceleration techniques. Therefore, further efforts are needed to explore the role of alternative methods, like using pressure when packing the geopolymers, to enhance the strength of, and decrements in, alkali activator amounts.

Various studies incorporated heated pressured catalysis (PC) and reported high strength results by enhancing the wettability of precursors [16,17]. The applied pressure removes the voids and brings all particles in close contact with each other, and the temperature helps in leaching the constituents for reactivity. An FA-based geopolymer was cast at 350 °C under pressures varying from 13.8 to 41.4 Mpa, which resulted in a high strength gain (80–130 Mpa) [17]. Similarly, warm pressing by applying 200 Mpa pressure at the temperature of 130 °C for one hour gave a strength of 149 Mpa [18]. This approach has been applied to conventional concrete and rubberized concrete, and was described in that study as a “compression casting approach” where recycled aggregate concrete was compressed and promising results were obtained [19]. However, heating and warming, along with the pressure, may not be a sustainable option and, if carried out separately, there may be more rapid production, along with less utilization of energy. 

Furthermore, adding crystalline particles (fillers with surface compatibility with the precursor) to FA-based geopolymers is another approach that is receiving great interest in the ongoing research, e.g., clay. These efforts are ultimately aimed to reduce the amount of alkali activators, which are hazardous, corrosive, expensive, and not environmentally friendly. Clay is one of the most common crystalline materials used in the construction industry after its calcination at 700–1100 °C, as it only becomes reactive/pozzolanic at high temperatures, not in ambient conditions [20]. This makes clay an environmentally non-sustainable option [21]. Clay has also been used in the development of geopolymers and is considered a pioneering precursor for the development of geopolymers, where strength is obtained at a lower temperature (200 °C). Nonetheless, it is also true that its particles are plate-like angular structures and capable of bearing load [7]. Owing to this property, it may improve the microstructure of the FA-based geopolymer and may contribute to strength gain without high-temperature calcination. Evaluating this is one of the main contributions of this study to the existing literature. 

On the other hand, sand is another material that has been extensively used in the construction industry to formulate concrete or mortar [22]. While dune sand (DS) is a rejected sand in the construction industry, its fineness and poor grading are the main barriers to its utilization in concrete and mortar [23,24,25,26]. However, it has a range of granule sizes, high silica content (55–85%), and lesser adhesion; these properties may be used for the strength enhancement of geopolymers and make it an ideal material to be utilized as an aggregate or filler by customizing its granularity [27]. It is currently being used in research studies on cement-based mortars and concrete [28,29]. Recent research has highlighted the under-addressed features and challenges regarding the utilization of DS in geopolymers [27]. Moreover, a huge range of industrial, commercial, and organic wastes have been incorporated with FA to add to its existing properties [30,31]. DS is an inorganic waste that still needs to be investigated further [32]. This will help minimize to the harmful impacts that desert waste pose to the ecological environment. Moreover, DS may also help to reduce the cost of geopolymers with a higher strength, as its low pH may also reduce the amounts of alkali activators that are needed.

**Figure 1:**
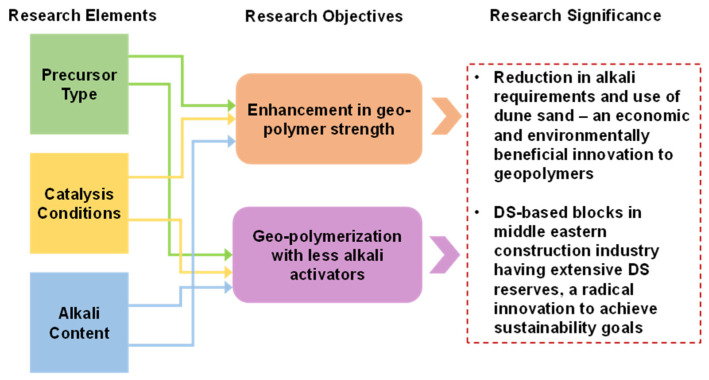
The incorporation of existing research elements to achieve the objectives of stronger and more sustainable geopolymers using a novel material and technique.

In this study, the improvement in the strength of FA-based geopolymer is achieved by reducing the alkaline activator through employing the PC technique. Furthermore, with the intention of further increasing strength and lowering the A/P, clay and DS were incorporated into FA-based geopolymer. The compressive strength was evaluated as the decisive property of the developed geopolymers. The attained strength is verified at the molecular and micro levels by conducting FTIR and SEM analyses, respectively. Moreover, a schematic model for the attainment of strength was developed. Furthermore, the sustainability of the geopolymer was quantified by evaluating environmental and economic indices. The novelty of this study is that it highlights ways to utilize clay other than calcination and sustainable ways of catalyzing the strength gain mechanism and proposes DS utilization in the geopolymers industry. The significance of the work, along with the research elements and objectives, are described in Figure 1.

## 2. Methodology

### 2.1. Materials

**Figure 2:**
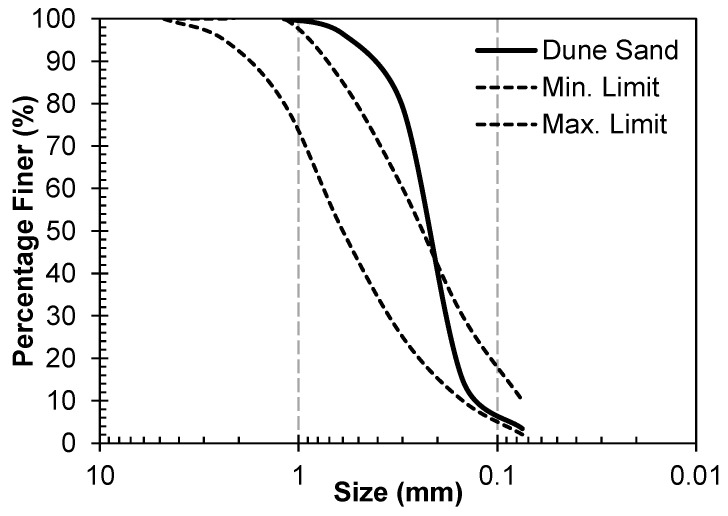
Gradation curve of dune sand used in this study and its comparison with ASTM limits for fine aggregates.

Fly ash (FA) was used as the main source of amorphous alumino–silicate material. It was obtained from the coal power plant in Sahiwal, Pakistan. The XRF analysis of the FA is presented in Table 1, and it satisfies the Class F criterion according to ASTM guidelines [33]. A particle size distribution of the FA was presented by the author’s research group [34], and D_50_ was 3.10 μm. The clay was used as a filler material and the source of SiO_2_; it was obtained from the brick manufacturing company, its particle size was measured by hydrometer [35], and the size varied from 1 to 57 µm. The third solid material used in this work was dune sand (DS), which was obtained from the Thal desert of Pakistan. It is light brown, and its fineness modulus, specific gravity (g/cm^3^), and apparent density (g/cm^3^) were 1.80, 2.21, and 1.72, respectively. It can be observed that its fineness modulus is significantly less than that of river sands (varying from 2.45 to 2.65) [22], which verifies its fineness. Its gradation curve, along with the ASTM limits, are shown in Figure 2 [36], and it can be observed that its D_50_ is 0.2 mm. The XRF analysis of clay and DS is also presented in Table 1.

The combination of sodium silicate (Na_2_SiO_3_) and sodium hydroxide (NaOH) was used in this work as an alkaline activator. Both materials were purchased from the local market. The Na_2_SiO_3_ was available in liquid form, with 55% and 45% solid Na_2_SiO_3_. Its modulus (SiO_2_/Na_2_O) was 1.2, whereas NaOH was available in white pallet form, and it was 98% pure. The NaOH solution of 12 molar concentration was prepared in the laboratory using deionized water. This was a highly exothermic reaction; therefore, the solution was prepared 24 h before mixing with the precursor.

### 2.2. Specimen Preparation

Specimens were prepared for different factor investigations in four phases: first, the SS/SH ratio and curing temperature, then the A/P ratio and curing temperature, the PC technique, and, lastly, the replacement of FA with clay and dune sand. In the first phase, the mix was prepared with FA, a fixed A/P, and curing condition, but with varying SS/SH. Next, the A/P was reduced and curing conditions varied while SS/SH and FA were constant factors. In the third phase, the A/P ratio was reduced even further, and the specimen preparation technique was replaced with PC. Lastly, the FA was replaced with clay and DS in percentages by weight.

#### 2.2.1. Na_2_SiO_3_/NaOH Variation

FA (precursor) was used as a solid, and a combination of Na_2_SiO_3_ (SS) and 12 molar solution of NaOH (SH) was used as a liquid (alkaline activator), and geopolymer paste was prepared by maintaining the alkaline-to-precursor (A/P) ratio of 0.30. Three values of Na_2_SiO_3_/NaOH (SS/SH) were used (2.0, 2.5, and 3.0). All constituents were mixed in a high-speed mixer (245–850 RPM without any load) for approximately five minutes. Then, the paste was poured in layers in a steel cube of size 50 mm with adequate temping/compaction. Specimens were covered by polythene sheets and put in an oven at a temperature of 60 °C for 24 h. 

#### 2.2.2. Reduced A/P and Curing Variation

**Figure 3:**
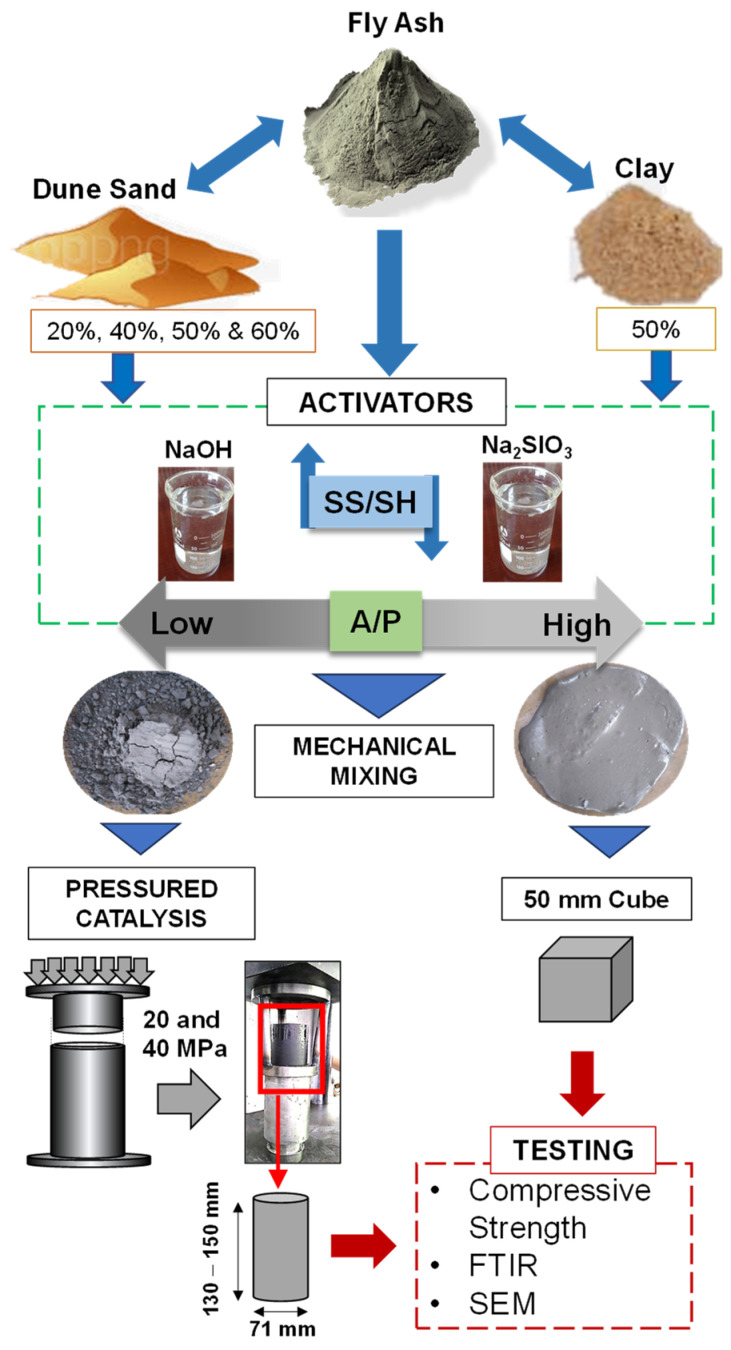
Schematic presentation of specimen preparation using a convention approach and the proposed pressured catalysis technique.

In the second phase of experimentation, the A/P was reduced to 0.25 and only 2.5 SS/SH was used for the casting of specimens. All specimens were prepared in a similar manner. However, a uniform slurry was not formed in this phase due to the low value of the A/P ratio. To evaluate the influence of curing temperature, the specimens were cured at ambient conditions (20 °C) and elevated temperatures of 60 °C and 100 °C for 24 h only. A summary of the specimen preparation is presented in Figure 3.

#### 2.2.3. Pressured Catalysis

In the third phase, mechanical activation was introduced, where the semi-dry mixture, by lowering the A/P ratio, was activated by applying pressures of 20 and 40 MPa. The specially designed cylindrical molds were used, where the piston moves inside the mold for the application of pressure to the paste. The internal diameter of the cylinder was 71 mm, and the height was 150 mm. It was filled with the paste and placed in a universal testing machine for the application of load. Upon reaching the 20 MPa value, the load was sustained for one minute and then released. The specimen was inverted and, by placing the tripod stand on its top, the load was again applied for de-molding. A schematic diagram of the mechanical activation is illustrated in Figure 3. Similarly, a 40 MPa activation load was applied, and both types of specimens were covered by a polythene sheet and placed in an oven for curing at 60 °C for 24 h.

#### 2.2.4. Incorporation of Clay/Dune Sand

To reduce the amount of FA, clay and dune sand were incorporated to develop geopolymer paste. The variation in clay amount varied from 0 to 100%, and the author’s research group reported a detailed investigation of the mechanical strength without using a novel technique [6]. Only 50% clay and 50% FA (by weight) were extracted from that study, and efforts were made to further enhance the strength via pressured catalysis. The specimen was prepared using A/P = 0.25 and SS/SH = 2.5, and its mix was catalyzed by applying 20 and 40 MPa molding pressure.

For DS, no data are available on making the geopolymer paste. Therefore, DS was mixed with FA at three different dosages (20, 40, and 60 wt. % DS). In the trial specimen preparation, it was observed that, at a higher A/P ratio (0.25), a flowable slurry was formed and, during pressured catalysis, all liquid flowed outside. Therefore, the A/P ratio was reduced to 0.12–0.16, the amount of NaOH was also reduced, and SS/SH was set at 1.5. Afterward, specimens were cast by varying SS/SH from 1.5 to 2.5. Finally, 50% DS and 50% FA was prepared with an A/P of 0.16, where SS/SH was 2.5. The curing condition of FA–clay and FA–DS composites was hot-curing at 100 °C for 24 h.

### 2.3. Testing

The primary test carried out on all types of specimens (50 mm cubes or 71 mm diameter cylinders) was the compressive strength test, which was conducted according to ASTM C 39. Three specimens were used for the evaluation of each factor under investigation; their average value is reported as the compressive strength of the specimen, which was measured at 7 d. The strength enhancement was assessed at the micro level by conducting an SEM analysis for FA- and FA–clay-based samples. The images were taken at 500 X magnification using field emission Zeiss Sigma 500 VP SEM. After conducting a strength test, the crushed specimen was further ground to pass through sieve # 200. The powdered sample was analyzed for evaluation of the Si–O–T band shifting by Fourier transform infrared (FTIR) spectroscopy. The FTIR spectra were scanned from 500 to 4000 cm^−1^ wavenumber, and only 600–1500 cm^−1^ is presented.

## 3. Results and Discussion

### 3.1. Parameters Influencing the Strength of FA-Based Geopolymer

#### 3.1.1. Varying Na_2_SiO_3_/NaOH

**Figure 4:**
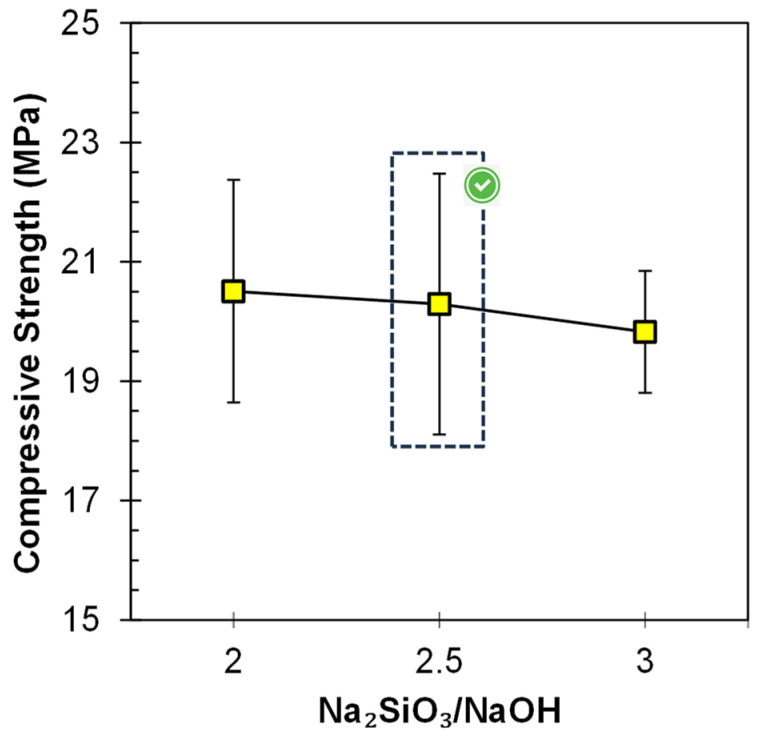
Influence of Na_2_SiO_3_/NaOH ratio on the compressive strength of FA-based geopolymer paste.

The influence of SS/SH on the compressive strength of the FA-based geopolymer paste is shown in Figure 4. It was observed that, at a constant value of A/P, i.e., 0.3, despite the increase in the ratio of SS/SH from 2.0 to 3.0, there is not much prominent variation in the compressive strength. Its value remains almost the same, i.e., about 20 MPa, for all the SS/SH ratios. This was due to the lower value of the A/P ratio; most of the reactive precursor seems to have been utilized by the activator. At a higher A/P ratio (0.5), there was an influence of SS/SH on the compressive strength and the strength increased, by increasing SS/SH from 2.0 to 2.5, from 27 MPa to 32 MPa [12]. However, the 2.5 value can be concluded as the optimized value, and afterward, no further increment in the strength was observed either by increasing the A/P ratio of SS/SH [12]. An increase in the SS/SH ratio, which is responsible for the increase in the amount of Na_2_SiO_3_, does not provide any facilitation for the acceleration of geo-polymerization so that mechanical strength improves [37], nor does it act as a hindrance due to the excessive silicate species causing a prominent decrease in the compressive strength [38].

#### 3.1.2. Varying Alkaline-to-Precursor Ratio and Curing Temperature

**Figure 5:**
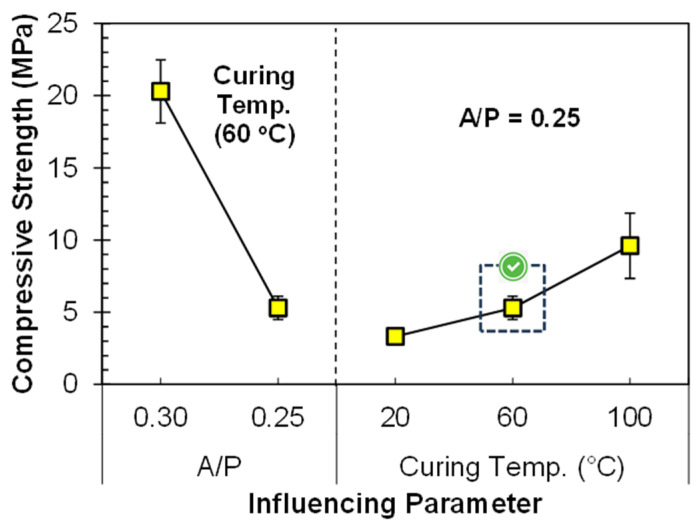
Influence of A/P ratio and curing temperature on compressive strength of FA-based geopolymer paste.

It is a well-known fact that the water-to-cement ratio affects the mechanical strength of conventional concrete. Similarly, the liquid-to-solid (A/P) ratio between activator and precursor content in geo-polymerization has a significant effect on mechanical performance. The influence of the A/P ratio on the compressive strength is shown in Figure 5. It is observed that, at 0.3 A/P ratio, the strength was 20.3 MPa, and after changing the A/P ratio from 0.30 to 0.25, the strength was reduced from 20.3 to just 5.3 MPa. The curing condition for each specimen was still constant, and it was 60 °C for 24 h. An increase in the A/P ratio, using an alkaline activator with the combination of NaOH and Na_2_SiO_3_, increases the SiO_2_/Al_2_O_3_ ratio of the system. Due to the increase in the SiO_2_/Al_2_O_3_ ratio there is an increase in the amount of Si species responsible for more Si–O–Si bonds. The Si–O–Si bond is much stronger than the Si–O–Al bond and is responsible for improving the compressive strength of the geopolymer [39]. Secondly, a higher A/P ratio ensures more water is available for the hydrolysis–polycondensation stage in geo-polymerization, ultimately improving the compressive strength.

In addition to the A/P ratio, curing temperature also plays an important role in enhancing the compressive strength. As the curing temperature was increased to 60 °C and 100 °C the compressive strength of specimens also increased, by 59.6% and 189.2%, respectively, as compared to the compressive strength of specimens cured at 20 °C (Figure 5). The increase in the compressive strength at 60 °C is due to the presence of a large portion of crystalline phases in FA that need a higher temperature to take part in chemical reaction, hence accelerating the geo-polymerization process [3,4]. A few studies also suggested that keeping the curing temperature higher improves the geo-polymerization process, and almost 70% of the geopolymer strength can be attained within less time [2,37].

#### 3.1.3. By Pressured Catalysis

**Figure 6:**
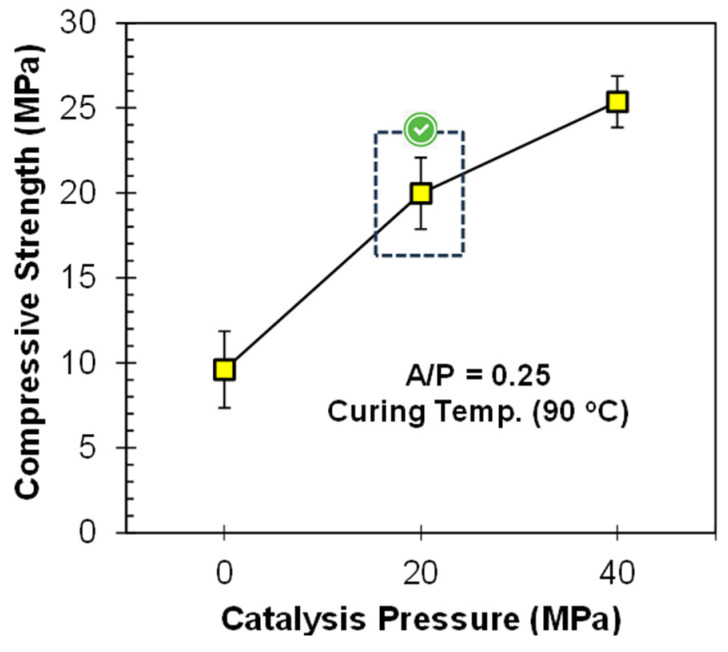
Influence of PC on the compressive strength of FA-based geopolymer paste.

The strength was further enhanced by mechanically activating the geopolymer paste. Therefore, a pressure of 20 MPa was applied during specimen casting, and the compressive strength results are presented in Figure 6. It was observed that the compressive strength value was only 9.6 MPa without applying any pressure. Via the application of pressure (20 and 40 MPa), the compressive strength was increased from 9.6 to 25.4 MPa. The reasons for the increase in strength are as follows: (i) The initial pressure removes entrapped air bubbles from the geopolymer matrix, resulting in a reduction in porosity and pore volume due to compaction [17,40]. (ii) Due to compaction, the particles come closer to each other, which increases their wettability with the activator, allowing them to play a more active part in the geo-polymerization process, resulting in a denser matrix and yielding more strength [16,41]. When higher pressures are applied, most of the entrapped air is removed until 20 MPa and a slight rearrangement of particles occurs, showing a marginal increase in compressive strength; hence, the extra load is wasted [34]. Therefore, it is concluded that 20 MPa pressure is sufficient to catalyze the strength improvement process of geopolymers at ambient temperature.

### 3.2. Incorporation of Clay

**Figure 7:**
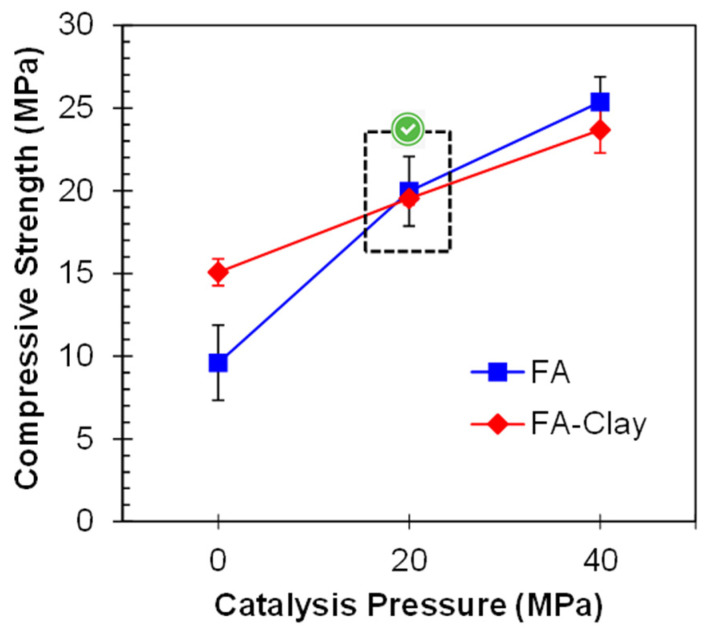
Influence of PC on the compressive strength of FA-Clay geopolymer.

The mix proportion of 50% FA and 50% clay has fulfilled several standards for the development of a pressed block or bricks [6]. Therefore, this mix proportion was used, and 20 and 40 MPa catalysis pressure was applied. The compressive strength of all specimens has been shown in Figure 7. The results show an exponential increase in the compressive strength with the application of pressure. According to Prasanphan et al., due to the application of pressure, the closeness of the particles increases, and they become more compacted. Due to this, their participation in the geo-polymerization process is improved [42]. By incorporating the clay, the optimum pressure is again 20 MPa, although some strength improvements were observed afterward. The reason for this might be the improvement role of clay in terms of compactness under high pressure, due to its plate-like particles [43]. Clay particles have an increased tendency to fit between the spherical shape particles of FA under the application of pressure. Similar results were reported by Khater and Ezzat for ground granulated blast furnace slag and metakaolin-based geopolymer prepared by the cold pressing method, in which 30–50 MPa was declared as the optimal pressure range [44]. The prominent decrease in compressive strength, along with a decrease in the proportion of clay content, especially for 40 MPa, provides evidence that clay behaves as a surface-active precursor, and hence takes part in geo-polymerization to some extent when cured at a high temperature of about 200 °C [7]. In another study, the strength of the specimens exponentially increases up to 20 MPa, i.e., 5.9 times, as compared to non-pressed specimens [7].

### 3.3. Incorporation of Dune Sand

**Figure 8:**
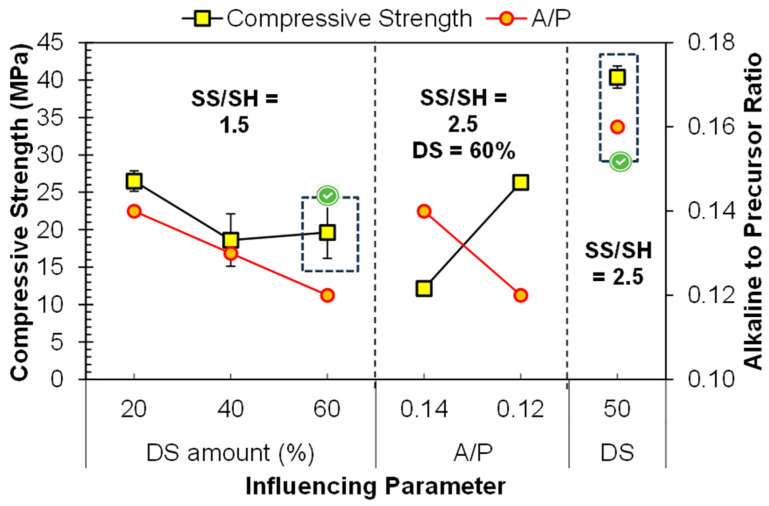
Parameters influencing the compressive strength of FA–dune sand composite geopolymer.

Dune sand (DS) is used as a precursor and filler simultaneously; its fineness and a large amount of silica content may contribute to the geopolymerization. As an initial trial, FA was replaced 20–60% DS, keeping the SS/SH ratio of 1.5, as DS contains a large amount of silica already. The compressive strength values are described in Figure 8. It can be observed that adequate strength (26.5 MPa) was obtained by using 20% DS, and it was decreased by increasing the amount of DS. The reduction in the strength was due to the reduction in active precursor, i.e., FA. The large crystalline part of DS resulted in a reduction in the strength. Moreover, the A/P ratio is also shown in the secondary *y*-axis of Figure 8. The A/P was set so that the semi-dry mix could bear the molding pressure. The reduction in strength was also due to the reduction in the A/P ratio [12].

With the intention of using more DS, the SS/SH was increased from 1.5 to 2.5, which contributes more Na_2_SiO_3_ and more SiO_2_/Al_2_O_3_. Very interesting results were obtained with a low DS content (20% DS): the strength was reduced by more than 50% (26.5 to 12.2 MPa) by increasing the SS/SH from 1.5 to 2.5. The reduction in the strength was due to the congestion of large amounts of SiO_2_ and Na^+^ in the matrix, which resist the geo-polymerization process [15]. However, for the case using 60% DS, the strength was increased from 19.6 MPa to 26.3 MPa by increasing SS/SH from 1.5 to 2.5, respectively. The increase in strength was due to the large SiO_2_/Al_2_O_3_, but the amorphous content of SiO_2_ was less, due to the lower amount of FA. Moreover, the surface activation of DS in the alkaline environment contributed to the strength, and therefore resulted in an increase in strength. 

Finally, to further improve the strength, the amount of DS was reduced from 60% to 50% and the A/P ratio was also increased from 0.12 to 0.16.; this resulted in significant improvement in the strength, and 40.4 MPa strength was obtained. An increase in the alkaline environment accelerates the dissolution process of precursors, increases the rate of geo-polymerization, and ultimately increases the compressive strength [4,7,12]. A correlation of the reactivity of the precursor with the mechanical strength has also been developed [3]. However, it is also evident that DS-based FA geopolymers provide an adequate strength, even at a lower A/P, compared to FA- and FA–clay-based geopolymers due to the occupying effect, as explained, along with the schematic model, in Section 3.4.

### 3.4. Schematic Model of Mechanism of Strength Gain

**Figure 9:**
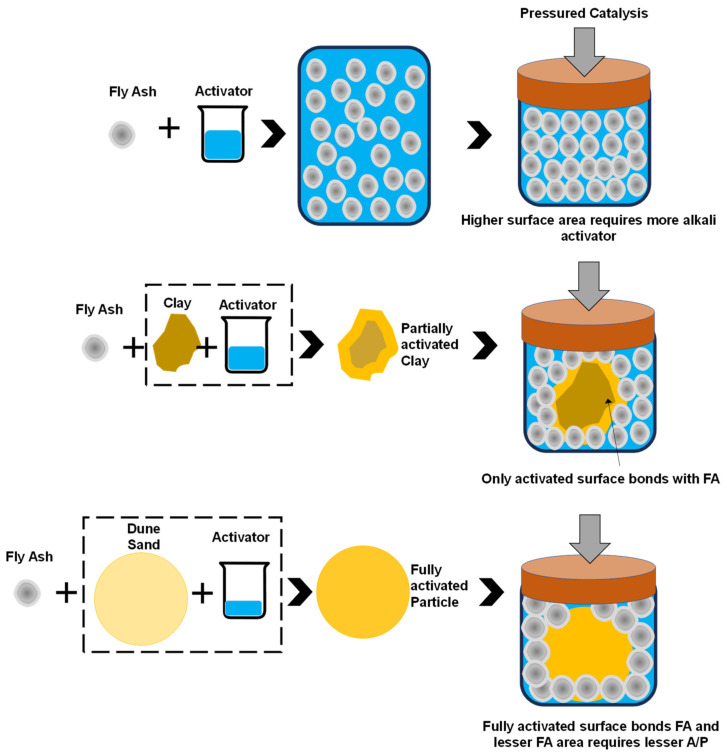
Schematic model for strength gain by FA and its composites under pressure catalysis.

The factors that influence the strength of the FA-based geopolymer with the addition of clay/DS is the fineness, the shape of the particle, and the adhesion between particles in an alkaline medium. It is evident from the particle size distribution that the particle size of FA was 3.10 μm, whereas the size of the clay and DS is 30 μm and 0.2 mm, respectively. The surface area of the FA is obviously larger, and since it is amorphous, it is dissolved in an alkaline medium and dissipates alkali and silica. After this, the polycondensation strength can be enhanced by the pressured catalysis. With applied pressure, the adhesion among particles increases and the whole surface of FA is dissolved, thus providing a dense structure and enhanced strength. The phenomenon is schematically shown in Figure 9. It can also be revealed from the SEM and FTIR analyses, as explained in Section 4. 

In the case of FA–clay composites, the clay particles are larger than FA, crystalline in nature, angular, and have plate-like structures. Their surface area is smaller compared to pure FA mix; however, being highly absorbent, it requires more activator. Furthermore, its crystalline structure did not activate fully; only the surface of the clay particles was activated. However, a compatible layer is formed between FA particles and clay, which contributes to the strength, as shown in Figure 9. Under pressured catalysis, the clay particles provide a dense structure and bears the molding load, the system becomes fully packed, and the strength further increases. 

Finally, in the case of FA–DS composites, both particles are spherical, but the DS particles are bigger in size and crystalline. This means that they have a smaller surface area as compared to FA and the reduced absorption capacity of DS particles resulted in drop in activator amount; therefore, the A/P was reduced to 0.16, as compared to FA as a single precursor and its composite with clay (where A/P was 0.25). The significant reduction in the A/P ratio was due to the occupying effect; the large particles (DS) occupied the solid volume within the porous and bulk volume of small particles (FA), and thus increased the packing density and, ultimately, the strength [45]. Furthermore, the sand particles bear the mechanical load and the adhesive layer consists of FA particles, which leach the silica and alumina and bond with the surface of the sand. FA, as a semi-crystalline material, establishes Octa–O–Tetra bonding. Silica and alumina species from the activator have non-bridged oxygen and are converted into bridged oxygen after polycondensation (Al–O–Si and Si–O–Si). In the case of crystalline particles (clay and DS), the Tetra–O–Tetra bonding was formed and thus contributed to the strength enhancement. A schematic model for the strength gain of FA, FA–clay, and FA–DS is illustrated in Figure 9.

## 4. Molecular and Micro Analyses

### 4.1. FTIR Analysis

**Figure 10:**
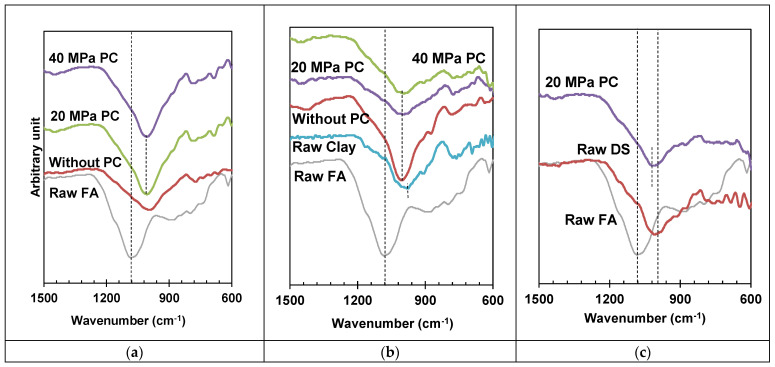
FTIR curves highlighting Si–O–T of FA and its composites (**a**) FA (**b**) FA-Clay composite and (**c**) FA-DS composite.

An FTIR analysis was performed to study the molecular behaviour of specimens under different mechanical activating pressures, as well as specimans with different composites of FA. A summary of raw and activated materials is shown in Figure 10. 

In the first case, where only FA was used, it was catalyzed by applying 20 and 40 MPa pressures. Figure 10a depicts all results; it was observed that raw FA has 1093 wavenumber cm^−1^; it was shifted towards the lower wavenumber and verified the process of geo-polymerization. With the application of pressure, the geo-polymerization process increased, revealed by the shift in wavenumbers from 1010.7 cm^−1^ towards the lower sides, i.e., 996.1 cm^−1^ and 995 cm^−1^ occurred upon the application of 20 and 40 MPa catalysis pressure, respectively. Although pressure enhances the geo-polymerization process, the influence of 20 and 40 MPa is marginal, and 20 MPa pressure can be optimized, as concluded in Section 3.1.3.

In the second case, the FA–clay composite was catalyzed at 20 and 40 MPa pressure. The FTIR spectrum is presented in Figure 10b. The raw spectrum of FA and clay is also shown, and a prominent shift in spectrum or the Si–O–T (T: Al or Si) band shift for FA can be observed, whereas this shift was absent in the case of the clay spectrum. It can be observed that the clay particle did not take part in the geo-polymerization, acted as the filler material, and helped to activate the geopolymer via its surface activation. Clay, due to its crystalline nature, acts as a load-bearing tiny block, which mingles in the geopolymer structure, whereas FA, which is amorphous in nature, acts as a cementing agent.

In the third case, where the composite was prepared by mixing DS with FA, the FTIR spectrum of raw and geo-polymerized specimens is shown in Figure 10c. Figure 10c is observed to be analogous with Figure 10b. Moreover, again, FA is shown to have amorphous silica, which actively participate in the process of geo-polymerization as compared to DS. Only Si–O–T shifting was observed for the FA specimen after geo-polymerization.

**Figure 11:**
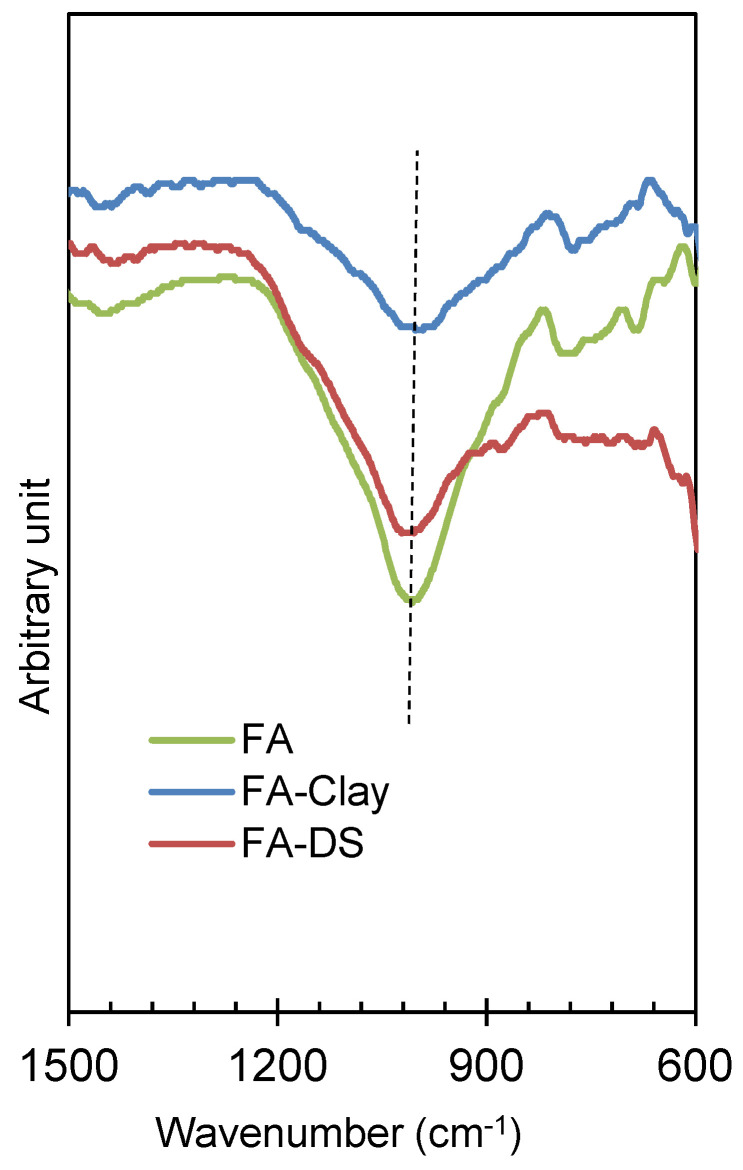
Si–O–T curves of optimized specimens of FA and its composites, activated at 20 MPa molding pressure.

Figure 11 describes the FTIR spectrum of the geo-polymerized specimens, more specifically highlighting the Si–O–T bands. It was observed that FA, as a precursor, is a highly amorphous material and has a sharp peak and has larger area under the curve, resulting in more geo-polymerization [6]. As compared to other composites, such as FA with clay and FA with DS, the same band was observed, revealing the same amount of geo-polymerization. However, 50% of the total FA was reduced by clay/DS. The pressured catalysis contributes to the same amount of geo-polymerization due to the surface activation of clay and DS materials. Furthermore, the crystalline and fine particles of clay and DS contribute to the molding pressure and enhance the mechanical strength, as explained in Section 3.2 and Section 3.3, respectively.

### 4.2. SEM Analysis

**Figure 12:**
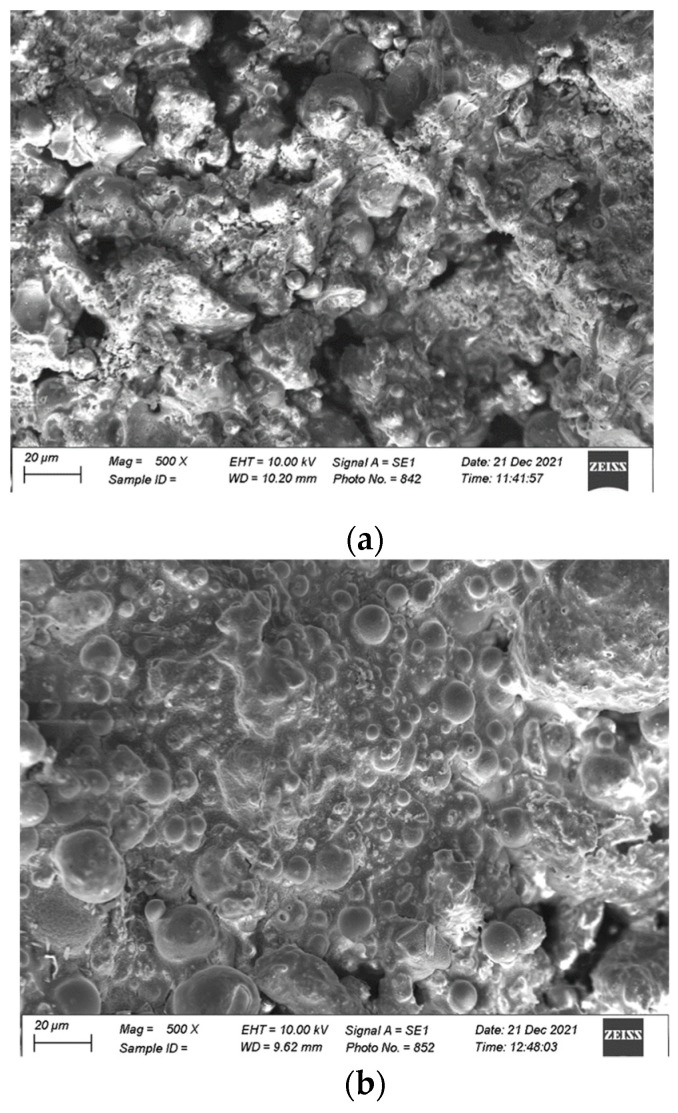
SEM analysis of FA-based geopolymer (At 500×, scale is 20 µm): (**a**) 20 MPa pressure and (**b**) 40 MPa pressure.

An SEM analysis was carried out, and Figure 12 presents the microscopic images of the FA-based geopolymers catalyzed at 20 and 40 MPa pressure. FA is amorphous and dissolves in the alkaline environment; therefore, no sharp spherical particles can be visualized in Figure 12a. The chemosphere is dissolved and a gel-like structure is observed, although some voids can also be seen in Figure 12a. However, such voids are absent in Figure 12b due to the catalysis of the specimen at 40 MPa. Higher pressure further densifies the paste and the alkaline solution fully reacts with the FA particles; therefore, the mechanical strength is increased, as explained in Section 3.1.3.

**Figure 13:**
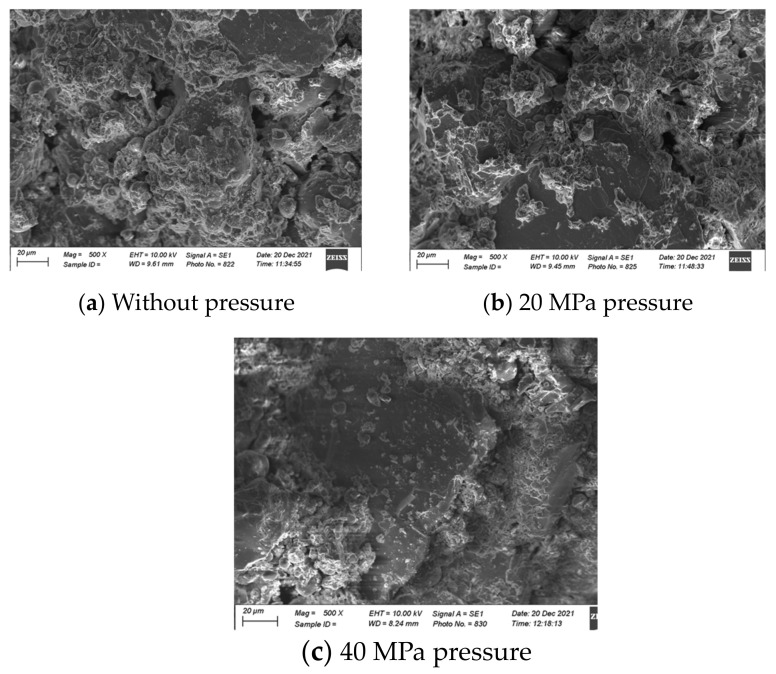
SEM analysis of FA–clay composites (at 500×, scale is 20 µm).

Figure 13 presents an SEM analysis of the FA–clay composites and compares the influence of mechanical activation. The sharp-edged plate-like structure is formed of clay particles that are crystallized and not dissolved in the alkaline environment. The sharp edges are clear in Figure 13a due to the absence of any pressure, meaning that voids can also be observed; thus, the structure has a lower mechanical strength. With the application of pressure, the surface of the clay particles was activated, and voids diminished, which resulted in an increase in the strength. Furthermore, the surface activation is clearer in Figure 13c.

## 5. Sustainability Assessment

Three pillars of sustainability are quantified: social, environmental, and economic. The social factor is linked to the strength of the block. The higher the strength compared to the standard or requirement, the higher the degree of social acceptance. However, the environmental factor was quantified for a block based on the CO_2_ emissions, and the economical factor was quantified based on a summation of the cost of the constituent raw materials. The size of the block considered for calculations was 220 × 110 × 75 mm. The details of the environmental and economic factors of the developed geopolymer blocks are described in the following subsections.

### 5.1. Environmental Factor

**Figure 14:**
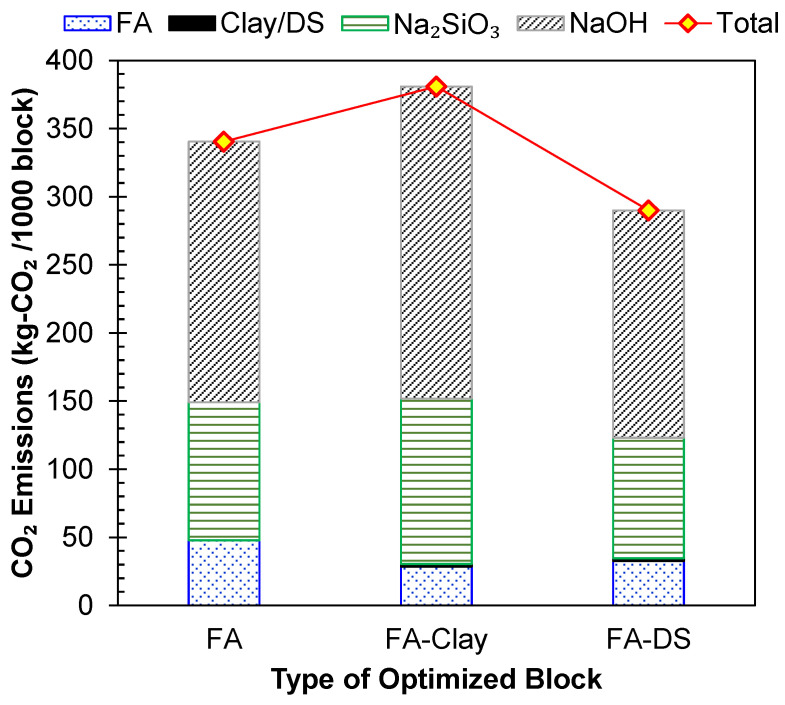
Quantification of environmental factors based on carbon dioxide (CO_2_) emissions of geopolymer blocks (Note: size of blocks is 220 × 110 × 75 mm).

To assess the sustainability of the three types of geopolymer blocks, FA, FA–clay, and FA–DS, their materials-based carbon emissions (CEs) were evaluated using the CE factor of the constituents, as presented in Table 2. The results are shown in Figure 14, and it was observed that FA–clay geopolymer blocks have the highest emissions, while FA–DS blocks had the lowest emissions to the environment, 23.9% and 14.8% lesser than those of FA–clay- and FA-based blocks, respectively. The major contribution to the emissions was the CE factor of NaOH, and Na_2_SiO_3_, while the FA, clay and DS had very low emissions. The activator amount in FA–clay was increased in the mix due to the inclusion of clay, which makes the FA–clay mix the highest-emitting geopolymer among the three. Whereas, in FA–DS, the activator amount is 36% less than the other two types, which is the main driving factor for the reduction in carbon emissions. Hence, the addition of DS is validated as an environmentally friendly measure to increase the sustainability of geopolymers. Closely similar results were reported in another study, where 50% DS was incorporated in a concrete mix, resulting in lowered emissions [46].

### 5.2. Cost 

**Figure 15:**
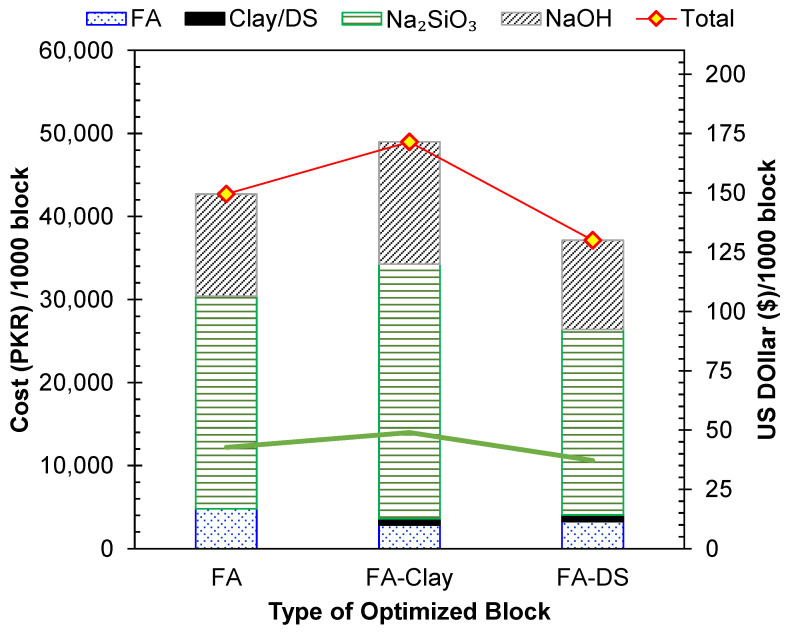
Per-unit cost of the three geopolymer types.

The second parameter used to assess the sustainability of the three types of geopolymer blocks was their materials-based cost. The per-unit cost for each was acquired from the local suppliers. The results, shown in Figure 15, reveal that the FA–clay-based geopolymers are the costliest option among the three, and the addition of DS lowers the cost by lowering the activator amount. The main controlling factor in terms of cost is the use of activators, as shown in Figure 15. Hence, the addition of DS can lower the cost of FA-based geopolymers by almost 13%, and costs can be lowered by 24% for FA–clay-based geopolymers. The addition of clay to FA increased the cost by 13%, due to the added activators in the FA–clay mix. A closely similar cost reduction of 20% was reported by bottom ash and water treatment sludge-based geopolymer bricks that were produced compared to conventional fired bricks [49]. The inclusion of DS is a cost-effective solution for geopolymers due to its low price, as it is a naturally occurring waste material, and it also reduces the requirement of an alkali activator, which is a costly constituent in geopolymers. Hence, DS has a strong potential to enhance the economic sustainability of geopolymers.

### 5.3. Sustainability Performance

To determine the final performance of each block compared to conventional fired clay brick, three sustainability parameters were considered: social, environmental, and economic. In the social category, strength was considered as the main factor in social acceptance. For environmental and economic performance, indices were quantified in terms of ratio of compressive strength (*f_c_*′) to carbon emissions (CEs) and cost (C), as given in Equations (1) and (2).

(1) $$\mathrm{Environmental}\;\mathrm{Index}=(f_{c}^{\prime })/\mathrm{CE}$$

(2) $$\mathrm{Economic}\;\mathrm{Index}=(f_{c}^{\prime })/C$$

**Figure 16:**
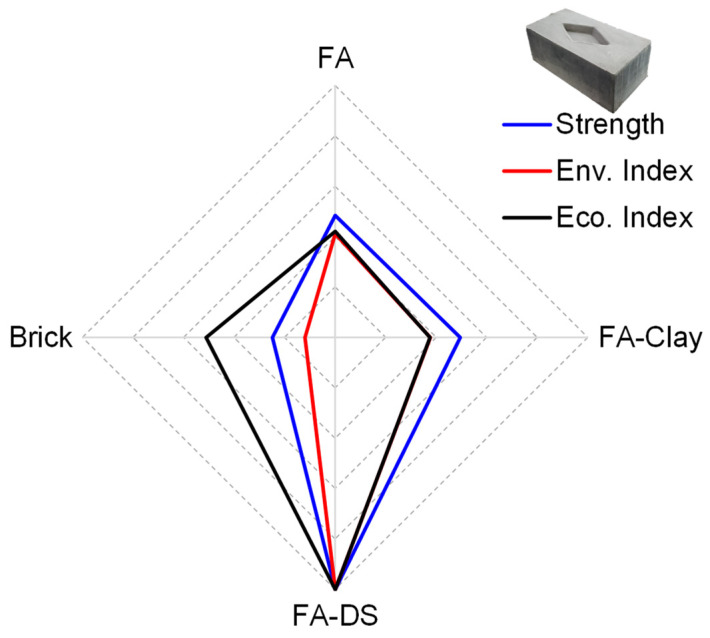
Sustainability performance score card of optimized geopolymer blocks.

The obtained results are listed in Table 3 and shown in Figure 16, where the innermost radar diagram represents the worst performance with the least social acceptance, i.e., the lowest strength value, and lowest values for environmental and economic indices. FA–DS blocks represent the best performance in all three parameters, with the highest strength, wide acceptance socially and in industry, and the highest environmental and economic indices. The FA–DS blocks provide around 60% better performance than FA and FA–clay blocks, and an 88% better performance than the conventional brick, environmentally. In terms of economic performance, the mixture yields a 57.9 and 62.2% higher economic index than FA and FA–clay, respectively, and 48.9% higher performance than that of brick.

**Figure 17:**
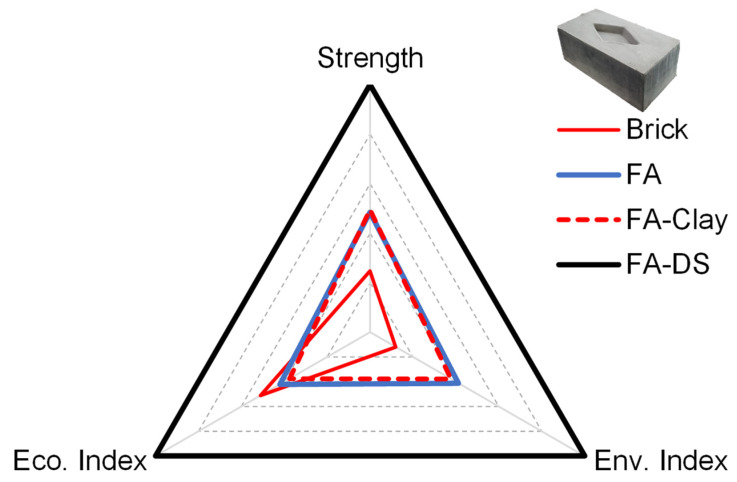
Performance against three pillars of sustainability.

The three pillars of sustainability are social, environmental, and economic, and their integration results in a sustainable block. In this work, strength is considered the social pillar; an appropriate strength leads to social acceptance. Other factors are economic and environmental, as shown in Figure 17. It can be observed that the FA–DS-based building block has high sustainability among all specimens, as well as conventional fired clay brick.

## 6. Conclusions

This work was designed with the intention of reducing alkaline activators by employing the pressure catalysis (PC) approach in geopolymers. The activator was further reduced by adding filler materials (clay and dune sand (DS)). The strength was discussed at the molecular and micro levels by conducting FTIR and SEM analyses. Finally, sustainability was quantified by estimating environmental and economic indices. The experimental work yielded the following conclusions:

1. The proposed pressured catalysis (PC) technique resulted in the enhancement of the mechanical strength of FA-based geopolymers by more than 100% as compared to the specimen with the same mix proportion but cast without employing PC.
2. The PC was optimized at a level of 20 MPa, resulting in compressive strengths of 20.0 MPa for FA, 19.5 MPa for FA–clay, and 40.4 MPa for FA–DS. Additionally, DS was produced at a lower A/P ratio of 0.16, and demonstrated twice the strength of its corresponding geopolymer specimens.
3. The addition of DS displayed a robust capacity to be utilized in geopolymers. Its uniform crystal particles play a substantial role in carrying the load during the PC and reduce the activator dosage due to their occupying effect.
4. FTIR analysis demonstrated that the Si–O–T bands shifted towards lower wavenumbers, confirming the geo-polymerization process of FA. However, the inclusion of clay/DS did not cause any additional decrease in the wavenumber. Their role in geo-polymerization was primarily related to their involvement in load-bearing by PC, surface activation, and lowering the alkali activator dosage.
5. SEM analysis showed that, due to PC, the FA specimen completely dissolves and actively participates in the geo-polymerization process. On the other hand, the crystalline particles (clay) bore the activation load, and their surfaces were activated in an alkaline environment. As a result, a dense microstructure was created, leading to an increase in mechanical strength.
6. Replacing 50% of FA with DS improves the sustainability by lowering the CO_2_ emissions by almost 15% and lowering the cost by 13%. Coupling FA with clay increases emissions by 10% and increases the cost by 13%.
7. The FA–DS geopolymer blocks enhance the environmental friendliness, economic viability, and social acceptance of existing FA geopolymers by more than 50%.

## Figures and Tables

**Table 1:** The XRF analysis of solid materials used in this work.

Materials	SiO_2_	Al_2_O_3_	Fe_2_O_3_	CaO	MgO	K_2_O	Na_2_O	SO_3_
FA	54.14	28.19	4.93	4.81	0.90	0.57	0.09	0.65
Clay	71.31	9.36	4.45	1.71	1.40	1.95	1.46	0.00
Dune Sand	67.43	11.83	6.53	4.70	2.08	1.46	2.44	0.02

**Table 2:** Carbon emission factor for raw materials.

Material	kg-CO_2_/Ton Emissions	References
FA	19.6	[47]
Clay	1.3	[46]
Dune sand	1.3	[46]
Na_2_SiO_3_	237	[48]
NaOH	1120	[48]

**Table 3:** Sustainability performance parameters score of geopolymer blocks and conventional fire clay brick.

Sustainability Parameters	FA	FA–Clay	FA–DS	Brick
Strength (MPa)	19.6	20.0	40.4	10.0
Env. Index (MPa/CO_2_ emissions)	57.5	52.5	139.4	16.7
Eco. Index (MPa/PKR)	0.5	0.4	1.1	0.6

**Figure A1:**
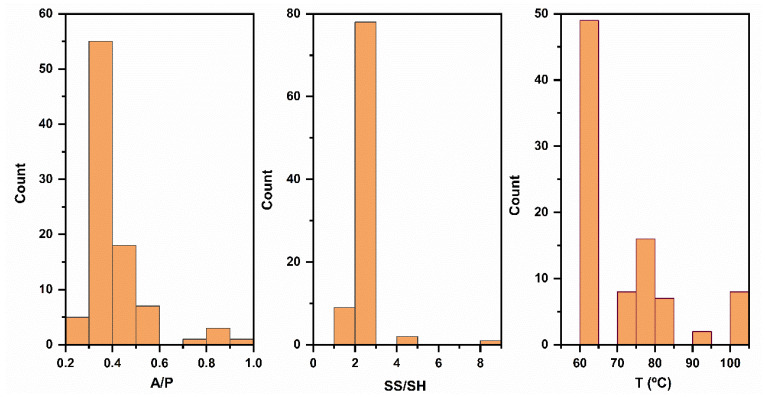
Histograms showing the frequency and range of alkaline-to-precursor (A/P), Na2SiO3/NaOH (SS/SH), and curing temperature (T °C) used for FA-based geopolymers.

## Data Availability

Data are contained within the article.
